# The lived experiences of a COVID-19 immunization programme: vaccine hesitancy and vaccine refusal

**DOI:** 10.1186/s12889-022-12632-z

**Published:** 2022-02-14

**Authors:** Nee Nee Chan, Khang Wei Ong, Ching Sin Siau, Kai Wei Lee, Suat Cheng Peh, Shakila Yacob, Yook Chin Chia, Vei Ken Seow, Pei Boon Ooi

**Affiliations:** 1 Department of English and Education, Faculty of Social Sciences, Quest International University, 30250 Ipoh, Perak Malaysia; 2 Faculty of Social Sciences & Liberal Arts, UCSI University, 56000 Kuala Lumpur, Malaysia; 3 Department of Pharmacology, Faculty of Medicine, Quest International University, 30250 Ipoh, Perak Malaysia; 4 Centre for Community Health Studies (ReaCH), Faculty of Health Sciences, Universiti Kebangsaan Malaysia, 50300 Kuala Lumpur, Malaysia; 5 Department of Pre-Clinical Sciences, Faculty of Medicine and Health Sciences, Universiti Tunku Abdul Rahman, 43000, Kajang, Malaysia; 6 Centre for Research On Communicable Diseases, Universiti Tunku Abdul Rahman, 43000, Kajang, Malaysia; 7 Jeffrey Sachs Center On Sustainable Development, Sunway University, Bandar Sunway, Malaysia; 8 Department of Medical Sciences, School of Medical & Life Sciences, Sunway University, 47500 Bandar Sunway, Selangor Malaysia; 9 Department of History, Faculty of Arts & Social Sciences, University of Malaya, 50603 Kuala Lumpur, Malaysia; 10 The International Institute of Public Policy and Management (INPUMA), University of Malaya, 50603 Kuala Lumpur, Malaysia; 11 Department of Primary Care Medicine, Faculty of Medicine, University of Malaya, 50603 Kuala Lumpur, Malaysia; 12 Emergency Medicine Department, Sunway Medical Centre, 47500 Bandar Sunway, Selangor Malaysia

**Keywords:** COVID-19 Immunization Programme, Lived Experiences, Vaccine Hesitancy, Vaccine Refusal, Malaysia

## Abstract

**Background:**

The COVID-19 pandemic has resulted in a global health emergency and lock-down measures to curb the uncontrolled transmission chain. Vaccination is an effective measure against COVID-19 infections. In Malaysia amidst the national immunisation programme (NIP) which started in February 2021, there were rising concerns regarding the prevalence of vaccine hesitancy and refusal, and therefore, vaccine uptake among Malaysians. Although there are many quantitative studies on COVID-19 vaccination, the subjective experience of individuals was understudied. This study aims to explore the lived experiences of Malaysians regarding vaccine hesitancy and refusal, and facilitating factors that could enhance vaccine acceptance and uptake.

**Methods:**

This qualitative study employed the hermeneutic phenomenological study design. Purposive sampling strategies were used to recruit Malaysians that had direct experiences with friends, family members and their community who were hesitating or refusing to accept the COVID-19 vaccines. A semi-structured interview guide was developed based on the expert knowledge of the investigators and existing literature on the topic. A series of focus group interviews (FGIs) was conducted online facilitated by a multidisciplinary team of experts. The group interviews were transcribed verbatim and analysed.

**Results:**

Fifty-nine participants took part in seven FGIs. We found that “incongruence” was the overall thematic meaning that connected all the 3 main themes. These themes comprise firstly, the incongruence between the aims and implementation of the National Immunization Program which highlighted the gap between realities and needs on the ground. Secondly, the incongruence between Trust and Mistrust revealed a trust deficit in the government, COVID-19 news, and younger people’s preference to follow the examples of local vaccination “heroes”. Thirdly, the incongruence in communication showed the populace’s mixed views regarding official media and local social media.

**Conclusions:**

This study provided rich details on the complex picture of the COVID-19 immunization program in Malaysia and its impact on vaccine hesitancy and refusal. The inter-related and incongruent factors explained the operational difficulty and complexity of the NIP and the design of an effective health communication campaign. Identified gaps such as logistical implementation and communication strategies should be noted by policymakers in implementing mitigation plans.

## Background

As of 9 December 2021, there were a total of 271.9 million people infected by the severe acute respiratory syndrome coronavirus 2 (SARS-CoV-2) globally. In Malaysia, a total of 2,707,402 cases were reported and more than 31,000 lives have been claimed by the COVID-19 pandemic [1]. This highly contagious infection has resulted in worldwide social distancing and lock-downs to curb the uncontrolled transmission chain [2]. Currently, there is no approved drug-based therapy available to cure the COVID-19 infection [3]. Hence, the success of ending the COVID-19 pandemic, or at least achieving the “herd immunity” [4], largely rests on vaccination. Recent data showed that there are as many as 8 vaccines approved and currently in use all around the world [5]. In Malaysia, five vaccines were approved by the Malaysian Ministry of Health namely: Pfizer-BioNTech BNT162b2, Oxford-Astrazeneca AZD1222, Sinovac CoronaVac, CansinoBio Ad5-nCoV and Sputnik V Gam-COVID-Vac [6]. However, the emergence of new COVID 19 variants such as the DELTA [7] and OMICRON [8] strains suggest that the original purpose of achieving herd immunity may never be achieved. As such, expectations of the efficacy of the COVID 19 vaccines would have to be managed and such communication is disseminated to the public.

The Malaysian National COVID-19 Immunization Program (NIP) was launched by a special committee known as Jawatankuasa Khas Jaminan Akses Bekalan Vaksin COVID-19 (JKJAV) in February 2021 and aimed to be “run smoothly, safely, effectively and in an orderly manner in the effort to curb and end the COVID-19 pandemic” [6]. It comprises three stages: Phase 1 target frontliners comprising public and private healthcare personnel, essential services, defence and security personnel; Phase 2 prioritizes senior citizens (those aged 60 and above), high-risk groups with chronic diseases such as heart disease, obesity, diabetes and high blood pressure, and people with disabilities; while Phase 3 (the current phase which is expected to end by February 2022) gives priority to adult population aged 18 years and above. The aim was to ensure that at least 80% of Malaysia’s adult population receive vaccines by February 2022 to reduce the spread of infections, hospitalization and death. The COVID-19 vaccination is voluntary and is provided free of charge to all people living in Malaysia (citizens and non-citizens) [6]. By September 2021, the NIP program was fully taken over by the Ministry of Health Malaysia with the change in portfolio of the Minister of Health [9].

Despite the rapid advances in COVID-19 vaccine development, the free vaccines offered in Malaysia and the roll out for population aged 12–17 years since September 8. 2021, the ultimate goal to break the coronavirus transmission chain is highly dependent on the acceptance and uptake of people towards these vaccines, a vital element that has been complicated by mixed perceptions regarding the spread of the virus, the safety of the fast-tracked vaccines, wrong information received/read/disseminated in social media or online and access issues.

According to the World Health Organization (WHO), vaccine hesitancy refers to a delay in the acceptance or refusal of vaccines despite the availability of vaccine services. It is a complex and context-specific phenomenon that varies across time, place and vaccines [10]. In Malaysia, a study conducted by June et al*.* in August 2020 (before the availability of the COVID-19 vaccine) found an intended vaccine acceptance rate of 93.2% via the survey conducted [11]. Subsequently, the intended acceptance rate was reduced to 67% (December 2020) [12] and 83.3% [13] (June 2021) respectively. As of December 2021, a total of 78.2% of Malaysians were fully vaccinated and 4,430,656 of them have completed their booster doses [9].

The theoretical basis of vaccine hesitancy transpired in the 1990s when researchers endeavoured to depolarize the gulf of pro-and anti-vaccination beliefs [14]. Various health behaviour models have been developed to depict vaccination intention and the associated factors that influence the decision to accept vaccination, including the Theory of Planned Behaviour (TPB) [15] and the Health Belief Model (HBM) [16]. In our study, we chose to adopt the “3Cs” Behavioral Model developed by the Strategic Advisory Group of Experts (SAGE) on Immunization, a multidisciplinary working group of scholars and practitioners with the WHO [14], due to its overall fit with our context of the study.

The three elements proposed by SAGE in the “3Cs” Model include complacency, convenience and confidence. Complacency refers to individual perception of risks and values of vaccines. It is manifested when the self-assessed risk of vaccine-preventable diseases is low and a vaccination program is not deemed as a solution. In other words, complacent individuals are often reluctant to conform to regulations when they feel that the risks are negligible [17]. Subjective probability proposed by Tversky and Kahneman indicated that personal judgement on risk is situational and is based on current information [18]. Consequently, a lack of transparency in policymaking and misinformation from the media can potently induce complacency.

Convenience is a factor in which physical barriers like availability, affordability and willingness-to-pay, geographical accessibility, ability to understand (literacy) and attractiveness of immunization services impede the acceptance of vaccination. For instance, Luz et al*.* reported that the availability of vaccination on-site in a workplace positively affects vaccine uptake among working-age adults [19]. Bedford et al*.* argued that convenience includes physical barriers to vaccine uptake, instead of comprising only a psychological state of mind. Convenience therefore, should encompass factors such as lack of a vaccine offer, difficulty accessing immunisation clinics due to long distances, and lack of communication about vaccine programs [20].

Confidence is crucial to promote engagement between members of the public and the government and subsequently, is a predeterminant of public compliance [21, 22]. Transparencies on the availability of vaccines and the occurrence of adverse events after immunization is a key component to gain public confidence [23]. However, intensive coverage on the incidences of adverse reactions globally like anaphylaxis [24], thrombocytopenia [25], cerebral venous sinus thrombosis [26] and death [27] from COVID-19 vaccines by social media or local media may discourage people from being vaccinated. Adverse after-effects experienced by the vaccine recipients themselves or their close family members influenced the confidence in vaccines. Likewise, extensive reporting on vaccine administration errors such as inadequate dosing negatively impacted public confidence [28]. Trust or mistrust in social institutions is a key to public compliance with preventive measures developed during SARS (2003) [29], Influenza A/H1N1 [30] and COVID-19 [31]. Moreover, both theoretical and empirical literature showed that contemporary societies are built on very low levels of trust [32], precipitating a trust-deficient response to immunization programs.

In this 3Cs Model, communication is considered not as a specific determinant in vaccine hesitancy, but more as a significant tool for the success of any immunization program [10]. However, there was agreement that deficient or poor communication about vaccines (e.g. their safety and effectuality) by institutional authorities can contribute to vaccine hesitancy. Some individuals who were beset by the lack of news or influenced by fake news were then influenced to refuse or hesitate on their intention to vaccinate [33]. In 2020–21, there was a predominance of fake news and widely circulated conspiracy theories regarding the efficacy of vaccines [34, 35]. As of March 2021, a study identified 578 rumours and conspiracy theories related to COVID-19 vaccines from 52 countries [36]. Additionally, some studies demonstrated a significant association between social engagement and positive health behaviours [37, 38]. Hence, it may be necessary to re-evaluate this 3Cs Model to determine the role communication plays in COVID 19 vaccine hesitancy and vaccine uptake. This is the gap in knowledge that this study also aims to explore and determine the significance of communication in vaccine hesitancy in this COVID-19 pandemic.

A later, more complex matrix formulated by SAGE, the Vaccine Hesitancy Matrix depicted the contextual influences like socio-cultural, economic or political influences; individual and group influences and vaccine-specific issues that correspond to vaccine hesitancy [10]. This is a more context-specific model that acknowledged the importance of context, society and individual and group differences in the success of a vaccination program. Studies on vaccine hesitancy have shown the significance of context-specific factors such as risk perception, social norms, group dynamics and political ideology as important determinants of vaccine hesitancy and refusal [39, 40]. A recent study in Malaysia by Syed Alwi et al*.* showed that religious and cultural reasons contributed 27.6% of the hesitant respondents [13].

The widely accepted definition of vaccine hesitancy by the SAGE Working Group [10] has been contested as its 3Cs Behavioural Model and the Vaccine Hesitancy Matrix have been critiqued as inadequate to cover the complex issue of vaccine acceptance and uptake [20, 41]. The 3Cs Model of Complacency, Convenience and Confidence are arguably psychological states of mind or sentiments held by people during an immunisation programme [20]. Some researchers argue that this model of vaccine hesitancy is problematic as it places vaccine uptake within the individual’s control and situates responsibility on the individual even if a vaccination system does not successfully reach him or her [20]. Vaccine hesitancy and uptake may be due to a combination of social and behavioural factors. Studies have shown that access barriers are an important factor to economically disadvantaged children not receiving vaccinations as their families face financial and logistical challenges [42, 43]. An alternative model proposed by Thomson et al. focused on the root causes of the vaccine coverage gap: the 5 As Taxonomy: Access, Affordability, Awareness, Acceptance and Activation. Thus, this study aims to evaluate the 3Cs Model to determine if this model is able to capture the social and behavioural factors that determine vaccine hesitancy and vaccine uptake through the findings of this study.

In this study, the lived experiences of participants during the COVID-19 Immunization Program in Malaysia are investigated. Through the meanings extracted from these lived experiences, a fuller picture of vaccine implementation, vaccine hesitancy and vaccine refusal would emerge. The research questions in this study are as follows:

1. What experiences are associated with vaccine hesitancy and vaccine refusal during the COVID-19 vaccination programme in Malaysia?

2. What could make people who hesitate and refuse vaccines accept the COVID-19 vaccines in Malaysia?

## Methods

### Design

Hermeneutic phenomenological methodology, as informed by the philosophical underpinnings of Heidegger was used in the research design of this study. Heidegger proposed the concept of ‘Dasein’ with ‘Being-in-the-world’ as an essential component [44]. This lived world is different from the physical world: the latter can be investigated using the scientific method but the former requires the researcher to discover a way into the world to reveal that world to others. Hermeneutic Phenomenology has its own philosophical and theoretical approach along with a research methodology consistent with this theoretical framework [45]. It was most suitable to inquire into the meanings of such individuals’ ‘being-in-the-world’ as they exist in their social, political, historical settings. Heidegger’s approach was used in this study to reveal new insights into the participants’ experiences of the COVID-19 immunization program and, particularly into the phenomenon of vaccine hesitancy and refusal in Malaysia.

Reflexivity is important in the design and conduct of hermeneutical phenomenological research as it guides researchers to ‘bracket’ their biases and pre-suppositions [46]. During this study, there was a critical analysis of the research experience, and the relationships between the researchers, participants, and the research processes through briefings, debriefings held before and after each focus group interview.

### Setting and Sampling

This study was conducted in Malaysia and the multidisciplinary study research team was from disciplines such as Medicine, Psychology, Information Technology, Public Policy and Education. Data collection was from 17 to 26 June 2021. This period was during the National Immunization Program Phase 1 (priority groups: healthcare workers, senior citizens, high-risk groups with chronic diseases and people with disabilities) and Phase 2 (adult population aged 18 years and above (citizens & non-citizens).

Focus group interviews (FGIs) were used as the primary method of collecting the data as this method afforded advantages such as greater stimulation and new perspectives from the interaction of the participants [47]. The FGIs were conducted entirely online on the Zoom video-conferencing platform and were recorded and transcribed verbatim. Informed consent was given by the participants who were assured of confidentiality and anonymity for this study.

Purposive sampling strategies such as snowball sampling and expert sampling were used to recruit the participants. 59 participants took part in seven FGIs and the duration of the FGIs ranged from 90 to 150 min. 49% were university students and 51% of the participants had occupations ranging from administrative service, teaching, lecturing, government service, self-employment and professional services. There were 2 unemployed participants (3.2%) and 1 politician (1.6%). Table 1 has the demographic details.

**Table 1:** Socio-demographic characteristics of the participants (*n* = 59)

Total Participants (*n* = 59)					
**Date**	**Session**	**Gender**		**Ethnic**	**Profession**
		Male	Female		
**17th June 2021**	FGI 1	5 (8.5%)	4 (6.8%)	4 Malay (6.8%) 3 Chinese (5.1%) 2 Indian (3.4%)	2 Medical professionals (3.4%) 1 Principal (1.6%) 1 Politician (1.6%) 2 Professors (3.4%) 1 Lecturer (1.6%) 2 Professionals (3.4%)
**18th June 2021**	FGI 2	1 (1.6%)	5 (8.5%)	2 Malay (3.4%) 3 Chinese (5.1%) 1 Indian (1.6%)	1 Government servant (1.6%) 2 Lecturers (3.4%) 1 Teacher (1.6%) 1 Self-employed (1.6%) 1 Professional (1.6%)
	FGI 3a	3 (5.1%)	7 (11.8%)	5 Chinese (8.5%) 1 Indian (1.6%) 4 Indigenous (6.8%)	1 Government servant (1.6%) 2 Lecturers (3.4%) 2 Professional (3.4%) 5 Students (8.5%)
	FGI 3b	3 (5.1%)	5 (8.5%)	5 Chinese (8.5%) 3 Indian (5.1%)	1 Retiree (1.6%) 1 Unemployed (1.6%) 6 Students (10.2%)
	FGI 3c	3 (5.1%)	3 (5.1%)	1 Malay (1.6%) 3 Chinese (5.1%) 2 Indian (3.4%)	6 Students (10.2%)
**25th June 2021**	FGI 4	6 (10.2%)	3 (5.1%)	8 Malay (13.6%) 1 Chinese (1.6%)	1 Lecturer (1.6%) 5 Professional workers (8.5%) 1 Unemployed (1.6%) 2 Students (3.4%)
**26th June 2021**	FGI 5	2 (3.4%)	9 (15.2%)	1 Malay (1.6%) 3 Chinese (5.1%) 7 Indian (11.9%)	1 Professor (1.6%) 10 Students (16.9%)
**Total**		23 (39%)	36 (61%)	59 16 Malay (27.1%) 23 Chinese (38.9%) 16 Indian (27.1%) 4 Indigenous (6.9%)	59

The inclusion criteria were that participants had to have experiences with friends, family members and their community who plan to accept or refuse the COVID-19 vaccines. The reason for this was because it was difficult to recruit participants who plan to refuse the vaccination or who would proclaim themselves to be hesitating and take part in a focus group discussion. The majority of the participants did not have the COVID-19 vaccination at the point of the FGIs. In the course of the FGIs, the participants revealed themselves to be either positive about the need of vaccination or were vacillating about taking the vaccinations.

Through snowball and expert sampling strategies, many of the participants recruited were volunteers or leaders in churches, temples or mosques or active in their community organisations. Many recounted their active interaction with their family and community members especially on the vaccines and helping their elderly members to register for the vaccines. During the period of July–August 2021 of the FGIs, many participants revealed that there was a dearth of timely, official information on the effectiveness of the vaccines and that they and their community of friends and families were therefore, affected by the preponderance of fake news or lack of information on the NIP.

### Data Collection

Seven FGIs were conducted with 59 participants until idea saturation. A semi-structured interview guide was developed based on the expert knowledge of the investigators and existing literature on the topic. Three investigators conducted the FGIs with support from four other investigators. Briefings and de-briefings were held before and after each FGI to check for biases and to review the reflexive notes written. Table 2 has the major points of the interview guide.

**Table 2:** Interview Guide

Interview Questions	
1	What are your experiences with the COVID-19 vaccination program? Tell us about what you have experienced, heard or read about this immunization program
2	Some people in Malaysia are refusing the vaccines offered or hesitating to register. Why are they behaving in such a way? What are the reasons?
3	If we look at fake news and conspiracy theories that are spreading, why do you think some people believe such fake news?
4	What could make people who hesitate and refuse vaccines accept the COVID-19 vaccines in Malaysia?

### Data Collection

The transcribed group interviews were imported into NVivo V.12 to manage and categorize the data. van Manen’s three-step procedures were used to analyze the data [42]. First, the interview transcripts were read wholistically several times to understand the overall meanings of the texts. Second, there was concentration on phrases or significant statements that stood out in the text or which answered the research questions. Third, the detailed approach involved a careful inspection of the text sentence by sentence.

There was a continual evaluation of the data to establish similarities and differences with each unit of data analysis. In the interpretive process, Gadamer’s strategies of the ‘hermeneutic circle’ and ‘fusion of horizons’ were used as the texts were read as parts and re-read as the whole, to allow new meanings and viewpoints to emerge from these scrutinies [48]. This then enabled the emergence of clusters of ideas and concepts which formed the basis of themes and sub-themes.

### Ethical Considerations

This study was approved by the Ethics Review Board at the Principal Investigator’s affiliated university (SUREC 2021/042) and Sunway Medical Centre (007/2021/IND/ER). An information sheet explaining the study to the participants was given and participants’ informed consent was obtained before the start of the FGIs. Specifically, participants were informed that the interviews would be recorded, the collected data to be coded with participants’ pseudonyms to protect personal information and, they had the freedom to withdraw from the study at any time.

### Trustworthiness

To enhance the trustworthiness of the research, Lincoln and Guba’s definitions for the establishment of rigour in qualitative research were adhered to closely [49]. To ensure credibility, reflexivity was practised throughout the research process to put aside prior assumptions and experiences about the phenomenon. Interviewers were trained and experienced in conducting FGIs. There was engagement with the participants in setting up and conducting the FGI sessions and contact continued with transcript checking. This was followed by intensive engagement with the data. To support transferability, there were rich descriptions of participants’ experiences quoting their verbatim statements. Dependability was ensured by a clear audit trail indicating how analytic and interpretive processes were conducted. Confirmability was established by sharing the transcripts with the participants. The final theme clusters were decided based on several rounds of discussions among the researchers. The entire process of the study was conducted according to COREQ [50].

## Results

Incongruence emerged as the overall thematic meaning that connected all the 3 themes and 5 sub-themes of this study (Table 3). There was incongruence between the official aims and implementation, and multiple realities and needs on the ground; between trust deficit in the government and the health authorities and trust in local leaders; between the official media and local social media in communication.

**Table 3:** Participants’ Lived Experiences of the COVID-19 Immunization Program in Malaysia

Themes	Sub-Themes
1.**Incongruence between aims and implementation of the National Immunization Program**	1. The macro program aims vs micro context-specific implementation: Herd Immunity vs "What's in it for me?" 2. Systematic implementation vs realities on the ground: Access issues of registration and transport to vaccination centres
2.**Incongruence between Trust and Mistrust**	3.Trust in local vaccination heroes a.Social Media Influencers b.Local GPs and Nurses c.Community Heads 4.Trust deficit a.Lack of knowledge and trust b.Mistrust of vaccines c.Mistrust of politicians & the government
**3. Incongruence in Communication**	5. Official media vs local social media a.Singular reality vs multiple, constructed realities b.Fake news, conspiracy theories, personal beliefs c.Lack of targeted communication towards the youth and adolescents leading to complacency and “tidak apa” (cannot be bothered) attitudes

**Fig.1:**
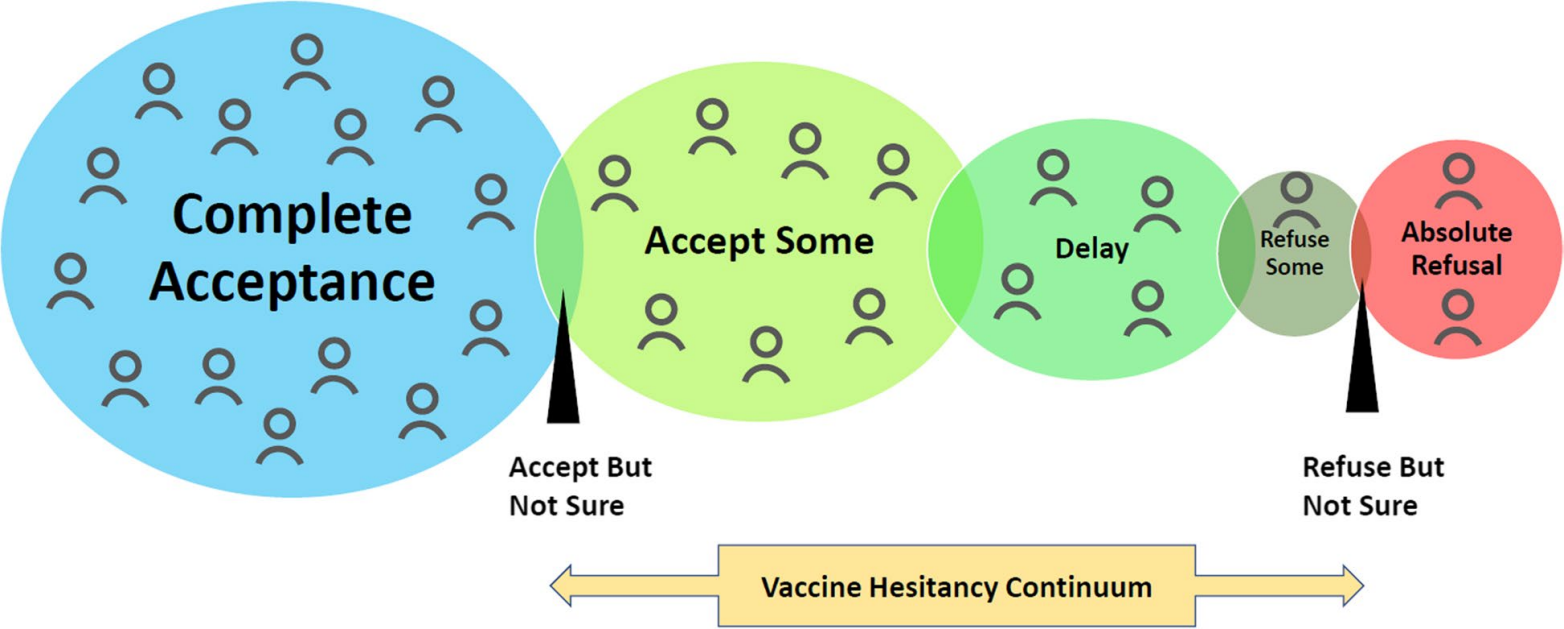
Vaccine Hesitancy Continuum.  represents demands.*(Adapted from Report of The SAGE Working Group On Vaccine Hesitancy)*

Vaccine hesitancy and refusal is a complex phenomenon that exists on a continuum between total acceptance, including high demand for vaccines, and absolute refusal of some or all vaccines (Fig. 1) [10]. The findings that emerged from this study show a complex picture of inter-related and incongruent factors from the responses of the participants towards the COVID-19 vaccination program and their attitudes towards the vaccines. In recounting their families’, friends’ and communities’ experiences, they described a range of vaccine hesitancy attitudes ranging from a lack of knowledge, inconvenience, mistrust in vaccines and mistrust of the prevailing government and health authorities.

### 1) Incongruence between aims and implementation of the National Immunization Program (NIP)

The aims and strategies of the NIP were to achieve ‘herd immunity’ and they were based on WHO’s recommendations and best practices from other countries [4]. Hence, the study which was conducted amid the NIP revealed incongruent outcomes between the aims and implementation. Many participants questioned the slow pace of the vaccination program, the initial, limited supply of the vaccines and the continual changes in the implementation protocols.

*“…the moment they heard the delivery was confirmed and coming, they released the second dose. For example, in our hospital, our doses were supposed to be during phase one… and then suddenly on the second week, we were told come, please bring forward all your 3rd and 4*^*th*^*-week recipients because we know doses are coming so we, we don’t need to reserve the second dose, so you know things are changing all the time”* (Participant W3, Medical Professional, FGI 1).

Some recounted experiences of their friends or families who were not convinced by the aim of achieving herd immunity. Implementation on the ground was thus, marred by this attitude and mindset of being complacent and waiting for others to carry the load. The participant below spoke about herself and other members of her community that thought alike.

*“I think there is this kind of mindset of wanting to piggyback on herd immunity. I suppose since the objective has been achieved, other people have already taken the risk on my behalf, so it doesn't seem crucial for me to do it, so if there’s no need to do it, then I don't do it”* (Participant G1, Unemployed, FGI 1).

#### b) Access issues of registration and transport to vaccination centres

The ‘orderly’ implementation planned by JKJAV was revealed to be chaotic in some areas as multiple realities impacted the actual implementation. The MySejahtera (My Wellbeing) mobile application was developed to assist in the management and mitigation of the COVID-19 outbreak [51] and it was used to register people for the NIP. It became apparent that the MySejahtera application had initial problems with logistics and deploying people to the right vaccination centres.

*“We are in Kajang but the first batch of elderly people were sent to vaccination recentres which is 30 km away from Kajang; people in Kajang had to go to Ampang Indah and some I also got to know were sent to Banting”* (Participant N1, Teacher, FGI2).

Additionally, due to logistical issues, some vaccination centres ended up with long queues of elderly and physically handicapped people who had to wait for long periods under the sun [52]. Rural and elderly citizens had problems using the mobile application due to a lack of technological competence. These groups also had problems going to the vaccination centres as they did not have the transport. There were accounts of missed vaccination appointments due to transport issues or fear of contamination from the big crowds at the vaccination centres. Hence, access issues posed a deterrence to these people. As such, these are systemic flaws which were exposed in the first and second phases of the NIP. As the quotation below shows, such physical and social obstacles could lead to anxious and fearful states of mind in the elderly, which could probably lead to their hesitation to take the vaccines.

“*Yeah, among the seniors..the other deterrent was even when they decided to (say) Ok, … and then other challenges that they encounter is not knowing how to register properly, not knowing how to do that properly um through the smartphone or never made it, did it, but didn't do it correctly and got deterred. Another thing is actually getting there, you know seniors can be afraid of having to go what they perceive as going alone to a strange place and to do something scary*” (Participant P10, FGI5).

### 2) Incongruence between Trust and Mistrust

#### a) Trust in local heroes and b) trust deficit in the government, COVID-19 news

What emerged from the findings was that although there was mistrust of the government’s management of the NIP, local politicians, the efficacy of the vaccines and their side effects, there also trusted in local vaccination heroes: the local doctors, nurses, community and village heads, the Instagram and YouTube local influencers. A lack of transparency in disseminating knowledge of vaccines, their efficacy and side effects and the progress of the NIP were cited as factors that contributed to vaccine hesitancy from February to June 2021 [53].

*“I’m saying transparency in terms of the delivery of the vaccine program: that people just say one thing, ok we were going to give this but people never sort of explaining it further, and people seem to don’t know what is happening, what is told and what is happening” (*Participant W3, Medical Professional, FGI 1).

Local politicians were perceived to be practising double standards in managing the COVID-19 pandemic with ordinary citizens fined for the transgressions of the standard operating procedures while politicians were perceived as escaping from these regulations [54]. As a result of these factors and arising from the trust deficit, participants recounted their experiences of people they knew and of themselves adopting a ‘wait and see attitude.’ Some refused the vaccines completely. These attitudes, however, were not immovable. Some experiences showed that elderly people changed their minds when they discussed the vaccines in detail with their doctors and nurses at the hospitals.

One of the participants, a young medical student recounted his grandfather’s journey towards vaccination. He tried to convince them with facts and evidence but to no avail.

*“..my grandfather didn't want to go to the vaccination so he just called me. Then the first question here is … will I be alive after the vaccination? ……So I really like uh cannot convince them to participate. But however, the KKM (Ministry of Health) people actually called them and explained to them that they have to (have)the benefits of getting vaccinated, so after that, he (was) actually convinced” (*Participant P8, Medical Student, FGI 5).

It appears that the grandfather was not convinced by his grandson who tried to persuade him with facts as he most likely did not trust these sources. He probably was convinced by the Ministry of Health personnel as he trusted them as a source of authority rather than his medical student grandson. Others said they would be influenced by the community leaders or social influencers that they trusted. Younger participants, in particular, said they would be influenced by their favourite local influencers or celebrities. Participants said that they were influenced by ‘influencers’ like Dr Amalina Bakri (Malaysian physician, Imperial College, London) and Douglas Lim (Malaysian comedian) who spoke about COVID 19 on their Tik Tok and Instagram videos. Some of the educated youth participants said they were influenced by younger politicians like Yeo Bee Yin and Syed Siddique whom they trusted.

*“I was reading … Yeo Bee Yin’s write up in a page about getting uh, why she (was) in her third or third or second trimester, (and) she decided, you know to take the Pfizer..and I think after that you know a lot of pregnant ladies started to take that. So, I think she's also quite a good influencer because she speaks with facts and data. And those who are educated, we.. are still sometimes hesitant some points, but when somebody has the data and dare to do (it) herself, you know, get vaccinated at her stage. So I think that helped a lot of people.* (Participant G19, Solicitor, FGD3a).

Malaysia has many context-specific issues, chief of which is that it is comprised of multi-ethnic and multi-religious groups with the Malays (69.8%), the Chinese (22.4%) and the Indians (6.8%) being the major groups with their multiple communities, cultural and personal beliefs [55]. Different ethnic groups in Malaysia were perceived to prefer certain vaccines. As the NIP stated that the people could not choose their vaccines, some people hesitated to take their vaccines.

*“… the Chinese believe the China technology, I don’t know lah, that’s is their point of view. I used to ask them why you want Sinovac. They say they believe (in) China technology because they want to work in China, they want to go China. That is their comment lah, I tend to ask them last time. The Malay prefer Pfizer because they can go to haji and umrah (pilgrimages to Mecca)” (*Participant M5, Medical Student, FGI 5).

Some vaccine refusals were because some Muslims perceived them as “not Halal”, that is, the vaccines were not permitted according to Islam. Elderly Muslims were motivated by their desire to go for their pilgrimages.

*“So, like for the elderly, we cannot go for pilgrimage in 2 years, so like their intention [to take vaccine] is only for going for the pilgrimage. Therefore, when they [elderly] are informed vaccine is required to go for pilgrimage, so they are motivated to get vaccinated”* (Participant F4, Unemployed, FGI 4).

There were accounts of how ethnic communities in Malaysia preferred to use home remedies to prevent COVID 19 infections.

*“..uh the Indians, they are more reluctant to get vaccinated because they believe in the traditional method you know this uh "asap" method eating "rasam", you know getting "kunya", then "kayu manis" lah and all this kind of thing, they think that because they are Indians’ and because they are eating a lot of spices because they are following the traditional method that they won't get Covid”* (Participant V3, Lawyer, FGD2).

### 3) Incongruence in Communication

#### Official media vs local social media

Communication has emerged as an important cause of vaccine hesitancy and refusal during this COVID-19 pandemic. In this COVID-19 outbreak, it can be argued that communication is as important as the other three Cs in influencing vaccine hesitancy and vaccine uptake. This is because, in this digital age of smartphones and proliferating technologies, about 86 per cent of the Malaysian population in 2021 are active social media users, highly engaged with consuming information and communicating with each other [55]. During the COVID-19 pandemic when official information on NIP was scarce and not timely, participants revealed that they and their communities educated themselves on the vaccines through the spread of information to each other. In particular, there was widespread use of WhatsApp, YouTube, and Facebook applications to disseminate information. Some participants recounted how some community members were influenced by conspiracy theories and fake news.

Health communication strategies adopted by the JKJAV like using the website, www.vaksincovid.gov.my and the Director-General of Health’s daily briefings were perceived as ‘too scientific’ and lacking in an emotional appeal to the general population. When compared to fake news that were proliferating, participants expressed that it was difficult to differentiate the facts from the fake news.

*“… so far, all the success stories are just science-based information and statistics. Even though they are factual, it doesn't have that powerful impact for most people. Because so what if there are millions of people benefiting from it, but if one person died from vaccination, and that person happens to be my family member? To me, it is 100% (impactful). So, people are having this dilemma of science-based (facts) but then (compared to) emotional (stories) that people died (from vaccination)”* (Participant G1, Unemployed, FGI3b).

There were differences in the way the different ethnic groups consume information and how messages were written and interpreted. In Malaysia, public perception was that different ethnic groups were influenced by information and news in their motherland countries, eg. Indians are influenced by news in India, Chinese by news in China. Vernacular newspapers were perceived as being more parochial in their treatment of community news [56].

*“I follow Malay newspaper, Chinese newspaper and other newspaper right, I think for me as a Chinese, I think Chinese media is very biased from the way that they talk like how they deliver the message….. But if I am not educated for maybe, my parents and you know all those aunties (and) uncles, they have the voicemail thingy, then it’s very scary. like whatever they say through the WhatsApp. So, the media role really play an important (role) because Malay newspaper, I don’t see that crazy, (it is) is only those many people who comment” (*Participant W2, Insurance Agent, FGD1).

Thus, in this COVID-19 pandemic, communication probably plays a significant role in developing vaccine hesitancy and refusal attitudes among the general population. Conversely, participants reported their families and friends who purportedly were vaccinated and became champions of the vaccinations through social media, and thus, influenced their peers and families positively.

## Discussion

The findings from this study are consistent with results from other studies prior to and during the COVID-19 Pandemic [13, 57, 58]. Explicating from the 3C Model of Vaccine Hesitancy [10], a lack of **confidence** in the effectiveness and safety of vaccines was a major determinant of vaccine hesitancy and refusal in this study. Additionally, from the participants’ lived experiences, there appears to be a high trust deficit in the JKJAV, the politicians who were policymakers, and the reliability of the healthcare system. As proposed by the Vaccine Hesitancy Matrix [10], contextual and issue-specific factors like personal, political and community belief systems added to the confidence or mistrust of certain brands of vaccines [13, 57].

**Complacency** was detected among the younger participants and their friends as they perceived risks of the COVID-19 disease as low, and vaccination was not deemed a necessary preventive action. This was probably because communication on the dangers of the virus was not directed to them as a specific group, and they assumed they were immune to the disease. We found that the attitudes of vaccine hesitancy towards the immunization program were not fixed. On the contrary, there appears to be the willingness to be counselled, and be provided with the correct information from their trusted leaders or heroes. A change of mind to embrace vaccination was deemed possible. This finding concurs with the results of other studies that reported peer effects on vaccination through various tools, such as imitation and information sharing [57, 59].

**Convenience** also emerged as a determinant of vaccine hesitancy and refusal in this study. The less digitally savvy sector of the community deemed the MySejahtera mobile application as cumbersome to use when attempts were made to register for vaccination appointments. Participants recounted how their community and family members were affected by access issues and this would affect their attitudes and trust in the NIP. These access issues involved logistics and mobility issues related to accessing the vaccination centres during the lockdowns. The lack of assistance and services together with poor communications undermined vaccine uptake [60]. Such access issues stemmed from systemic flaws in the administration of the NIP. Arguably, this factor of determining vaccine hesitancy in the 3 Cs Model may not be fit for purpose, as it places the responsibility of vaccine uptake and hesitancy on the individual where manifestly, the individual may have very little control on the social and physical factors that prevent him or her to get the vaccination [20].

**Communication** appears to be an important component to strengthen vaccine intent. In the Vaccine Hesitancy Determinants Matrix, communication and the media environment were proposed as contextual factors influencing vaccine hesitancy and refusal [10, 60]. Findings from this study reiterated the importance of communication, revealing that participants and their communities were inundated with misinformation in the form of fake news and conspiracy theories. In addition, there were incongruent influences such as the lack of timely information on the NIP, in particular, on the efficacy and safety of the vaccines [13].

The health communication strategies employed tended to rely on scientific facts and evidence, which probably failed with people who mistrusted biomedical research. Some studies also reported that logical and scientific evidence in health communication campaigns did not resonate with some individuals who were more influenced by the emotional appeals in misinformation [57, 59, 61].

### Limitations

The relatively small sample size and non-probability sampling of this study mean that the findings cannot be generalized. However, there can be transferability of the findings in other similar contexts, as the study results were consistent with other studies on COVID-19 vaccine hesitancy and refusal.

## Conclusion

From the themes that emerged from the participants’ lived experiences of the COVID-19 immunization program in Malaysia, it becomes apparent that there was incongruence between the official aims of the NIP and the realities, as well as needs on the ground. Paradoxically, while there was a trust deficit in the government and the health authorities, the people would believe their family members and local vaccination leaders. There was also incongruence in communication between the official media and local social media used by the people in their multiple settings.

This study describes and interprets the findings to reveal the complex picture of the COVID-19 immunization program in Malaysia and uncovers its impact on vaccine hesitancy and refusal. Hence, the significance of this study lies in its rich details of the phenomenon. Confidence, complacency and to some extent, convenience were found to be important determinants of vaccine hesitancy and refusal. We used the 3Cs Model to determine if the vaccine uptake and hesitancy attitudes during the NIP in Malaysia were based on Confidence, Convenience and Complacency. We found that while confidence in the vaccines and the health authorities did affect trust in the vaccines and the NIP, thus leading to accounts of vaccine hesitancy and refusal. Complacency was seen in the medical and postgraduate students’ accounts of how some of their peers and younger community members had an indifferent or ‘cannot be bothered’ attitude as they felt that since they were young and healthy, they would not be infected by COVID 19. Access issues were found to be an important deterrence to certain groups of people like the elderly, the disabled and the rural community. Hence, the factor of convenience does not adequately explain the institutional, social and physical factors that may influence vaccine uptake. The 3Cs Model would have to be updated to take into consideration such factors.

Communication and the media environment emerged as an important influence of vaccine hesitancy and uptake. In this twenty-first century, societies are beset by fake news and conspiracy theories through social and traditional media on an everyday basis. It is, therefore, important for health authorities to design effective communication campaigns to counter the misinformation. Context, group, individual and vaccine-related issues were also found to be significant determinants and should be factored into the design of health communication strategies.

This qualitative study can provide input to policymakers and program evaluators to develop appropriate strategies for immunization programs. Identified gaps such as logistical implementation and health communication strategies could be mitigated using training and capacity building in the health and community-based institutions. The present study also identified local culture, traditions and religion as determinants of vaccine hesitancy. which would thus, be useful to other Southeast Asian contexts which have similar settings. The greater significance of this study lies in its finding that communication probably plays a larger and more influential role in influencing vaccine refusal and hesitancy than in previous pandemics as the communication and media environment has changed irrevocably from previous decades.

## Data Availability

Data are available upon reasonable request from the corresponding authors.
